# Philadelphia chromosome-positive mixed-phenotype acute leukemia: a case report and literature review

**DOI:** 10.3389/fonc.2025.1623528

**Published:** 2025-07-25

**Authors:** Liqing Yang, Meijuan Huang, Yuanzhong Chen, Yong Wu

**Affiliations:** ^1^ Department of Hematology, Fujian Medical University Union Hospital, Fujian Medical University, Fuzhou, Fujian, China; ^2^ Fujian Provincial Key Laboratory on Hematology, Fujian Institute of Hematology, Fujian Medical University Union Hospital, Fuzhou, Fujian, China

**Keywords:** mixed-phenotype acute leukemia, *BCR::ABL1* fusion gene, tyrosine kinase inhibitor, case report, literature review

## Abstract

**Introduction:**

Mixed-phenotype acute leukemia (MPAL) is a rare type of acute leukemia with an incidence of less than 5% and Philadelphia chromosome-positive (Ph+) represents a distinct subtype.

**Case description:**

An 18-year-old female complained of recurrent fever with fatigue and chills for one month, and a week of growing lymphadenectasis. Bone marrow examination revealed two distinct populations of blast cells and the presence of *BCR::ABL1* fusion gene, leading to a diagnosis of Ph+ MPAL. The patient received induction chemotherapy of DVAP regimen combined with tyrosine kinase inhibitors (TKIs), and underwent allogeneic hematopoietic stem cell transplantation after achieving complete remission. To date, the patient has maintained sustained hematological and molecular complete remission.

**Conclusion:**

A literature review of 59 cases revealed that Ph+ MPAL is more common in adult, male patients and primarily manifests as B/myeloid subtype. Higher leukocyte counts and chromosome -7 abnormalities have been identified as poor prognostic markers. Acute lymphoblastic leukemia-type therapy is considered more effective for patients with MPAL, and in the TKI era Ph+ has become a subtype with a better prognosis.

## Introduction

Mixed-phenotype acute leukemia (MPAL) represents a rare type of acute leukemia with an incidence of less than 5% (1) and has a slight predominance in adult and male patients (2). Characterized by multiple lineage markers on a single blast population (biphenotypic leukemia) or single-lineage markers on distinct blast populations (bilineal leukemia) (3), MPAL was recognized initially in the 2001 World Health Organization (WHO) classification. Cases of bilineal and biphenotypic leukemia were grouped together and categorized into acute leukemias of ambiguous lineage (ALAL) according to the 2008 WHO classification (4). The diagnosis of MPAL is identified by a number of immunophenotypic markers based on the European Group for the Immunological Characterization of Leukemias (EGIL) or the WHO criteria (**Table 1**) (5, 6). Two large sample size retrospective studies on MPAL revealed that B/myeloid subtype accounted for 55-59%, followed by T/myeloid (33-35%), B/T (4-12%), and trilineage subtypes (0.9-2%) (7, 8).

**Table 1:** Comparison of two primary diagnostic criteria for MPAL.

Markers	EGIL 1995	WHO 2022
Myeloid Lineage		
MPO	2	YES
Lysozyme	2	If MPO-^a^
CD117	1	
CD13	1	
CD33	1	
CD65	1	
CD11c		If MPO-^a^
CD14	0.5	If MPO-^a^
CD15	0.5	
CD64	0.5	If MPO-^a^
Nonspecific Esterase		If MPO-^a^
B-Lineage		
cyCD79a	2	With CD19^b^
cyCD22	2	With CD19^b^
cyIgM	2	
CD19	1	YES
CD20	1	
CD10	1	With CD19^b^
TdT	0.5	
CD24	0.5	
T-Lineage		
CD3 cytoplasmic or surface	2	YES^c^ or Immunocytochemistry positive with non-zeta chain reagent
TCR αβ or γδ	2	
CD2	1	
CD5	1	
CD8	1	
CD10	1	
TdT	0.5	
CD7	0.5	
CD1a	0.5	

^a^At least two of these showing monocytic differentiation.^b^One of these if CD19 strong, at least two if CD19 weak (intensity does not exceed 50% of normal B cell progenitor by flow cytometry).^c^Intensity in part exceeds 50% of mature T-cells level by flow cytometry.

The pathogenesis of MPAL remains unclear, and the dysregulation of multiple lineage differentiation due to genetic and epigenetic heterogeneity might play an important role (9). A retrospective study of 39 patients with ALAL revealed that gene mutations were present in approximately 90% of patients, and genes involved in genomic stability and transcriptional regulation were frequently detected in the MPAL cohort (10). Gene mutations identified in MPAL are typically detected in acute myeloid leukemia (AML) or acute lymphoblastic leukemia (ALL) (11, 12). Genomic analysis revealed that *ZNF384* rearrangements were common in B/myeloid-MPAL, whereas biallelic *WT1* alterations were associated with T/myeloid subtype (13). Mutations in the putative chromatin modifier, PHF6, JAK-STAT and Ras signaling pathways are frequently observed in patients with B/T MPAL (14). Moreover, clonal chromosomal abnormalities (CAs) occur in 59%-91% of patients with MPAL (15). As two separate entities of MPAL, *BCR::ABL1* fusion gene induced by t(9;22)(q34.1;q11.2), also known as Philadelphia chromosome-positive (Ph+), was observed in 15%-30% of patients, especially in adults (8); whereas *KMT2A* rearrangement due to t(v;11q23.3) occurred more frequently in the pediatric cohort (16, 17).

Here, we report a case of a young patient with Ph+ MPAL who achieved complete remission (CR) after the first induction therapy involving the combination of tyrosine kinase inhibitors (TKIs) with the ALL-type regimen, and underwent allogeneic hematopoietic stem cell transplantation (allo-HSCT) with favorable clinical outcomes. Furthermore, we reviewed the relevant literature to further discuss the clinical characteristics and prognosis of patients with Ph+ MPAL.

## Case report

**Figure 1:**
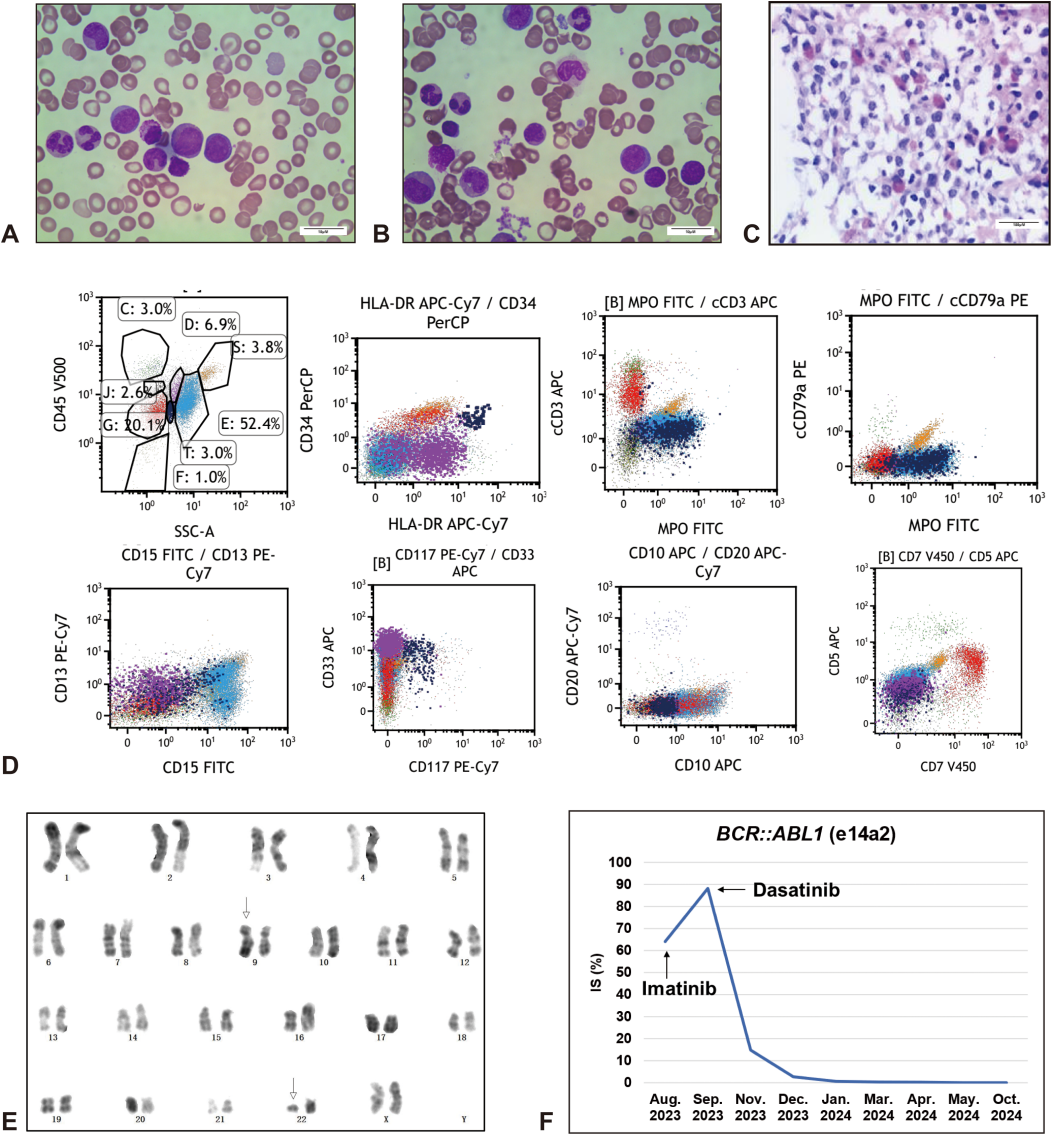
Paitent with Ph+ T/M-MPAL. Increased blast cells and eosinophils were evident in the peripheral blood smear **(A)** and bone marrow smear **(B)** (Wirght Giemsa, x1000, scale bar = 10 μm). **(C)** Bone marrow biospy suggested extremely active myeloproliferation (95%), with diffuse infiltration of myeloid blast cells and scattered primitive lymphocytes, marked eosinophilia, and myelofibrosis grade 2 (HE, x100, scale bar = 100 μm). Immunohistochemistry: MPO (+++), CD117 (-), CD34 (+), pax5 (-), CD3 (+), TDT(+), Lysozyme (+++), CD15 (+++), E-Cad (-), GPA (±), LEF1 (±). **(D)** Flow cytometry analysis showed two populations of blast cells by CD45/SSC gating, which were positive for T-lymphoid (cytoplasmic CD3, CD5, and CD7) (red, Group G, 20.1%) or myeloid differentiation (MPO, CD117, CD33, CD13, and CD15) (navy blue, Group T, 3.0%) markers, respectively. Group C, Lymphocytes; Group E, Granulocytes; Group D, Monocytes; Group J, Basophils; Group S, Eosinophils; F, Fragments and other cells. Data were acquired on a BD FACSCanto series flow cytometer (BD Biosciences) and analyzed using Kaluza Analysis software (Beckman Coulter). **(E)** Karyotype analysis by G-banding technique suggested a translocation between chromosome 9 and chromosome 22, t(9;22)(q34;q11), also known as Ph chromosome. **(F)** Dynamic analysis of *BCR::ABL1* fusion gene quantification. IS, International Scale.

An 18-year-old female patient was admitted to Fujian Medical University Union Hospital in Aug. 2023, who complained of recurrent fever with fatigue and chills for one month, and a week of growing lymphadenectasis. Physical examination showed an anemic appearance, multiple superficial lymphadenectasis and hepatosplenomegaly. The complete blood count (CBC) indicated evident leukocytosis (white blood cell [WBC] counts 127.68×10^9^/L), moderate anemia (hemoglobin 69.0 g/L), and normal platelet counts (223×10^9^/L). Lactate dehydrogenase (LDH) was markedly elevated at 888 IU/L. Enzyme-linked immunosorbent assay (ELISA) was weakly positive for mycoplasma and influenza B virus IgM antibodies, and a chest CT suggested infection in the middle and lower lobes of the right lung. Peripheral blood smear revealed 22% blasts together with 8.7% eosinophils (**Figure 1A**). Subsequent BM aspirates confirmed the occurrence of acute leukemia with 25.5% blast cells (**Figure 1B**), concomitant eosinophilia and secondary myelofibrosis (2+) (**Figure 1C**). Flow cytometry of the marrow aspirate revealed two populations of blast cells expressing T-lineage (cytoplasmic CD3, CD5, and CD7) or myeloid markers (MPO, CD117, CD33, CD13, and CD15), respectively (**Figure 1D**). Molecular studies involving gene mutations (**Supplementary Table S1**) and fusion genes (**Supplementary Table S2**) for leukemia identified a somatic mutation in *STAG2* (c.459_462 + 10delinsA) with a variant allele frequency (VAF) of 12.4% and *BCR::ABL1* fusion gene (e14a2). Karyotype analysis supported the presence of Ph chromosome (**Figure 1E**). Based on current evidence, the patient was diagnosed with T/myeloid MPAL with p210-*BCR::ABL1* fusion gene and *STAG2* mutations, then received DVAP regimen (daunorubicin [30 mg/m^2^/d ivgtt d1,8,15], vincristine [1.4 mg/m^2^/d ivgtt d1,8,15,22], cytarabine [100 mg/m^2^ q12h ivgtt d1-7], and prednisone [1 mg/kg/d d1-14, 0.5 mg/kg/d d15-28]) with imatinib (400 mg qd) for induction chemotherapy. The second bone marrow examination in Sep. 2023 revealed morphological CR with only 0.5% blasts, whereas the level of *BCR::ABL1/ABL1* mRNA increased from 64.07% to 88.19% international scale (IS) (**Figure 1F**), indicating a poor response to imatinib. After the subsequent replacement with dasatinib, the level significantly decreased to 14.84% in Nov. 2023. Considering the young age, sustained CR status, and the presence of adverse genetic abnormalities (*BCR::ABL1* and *STAG2*) for myeloid leukemia (18), the patient received six cycles of intrathecal chemotherapies consisting of methotrexate and cytarabine to prevent central nervous system leukemia (CNSL) and underwent 5/10 HLA-matched haploidentical allo-HSCT from her father in May 2024. The patient achieved deep molecular response (DMR) (*BCR::ABL1/ABL1* mRNA ≤ 0.01% IS (19)) and hematologic CR after transplantation, and has maintained sustained remission to date with regular follow-up.

## Literature review

Ph chromosome in adult patients is more common in MPAL than in AML, while the frequency is similar to that in ALL (15, 20). The presence of p210-*BCR::ABL1* in MPAL should raise suspicion for chronic myelocytic leukemia (CML) in blast crisis, with evidence of prominent splenomegaly and elevated granulocytes in prior history (11, 21, 22). In the present case, the diagnosis of blast phase CML cannot be excluded considering the classic characteristics of significant leukocytosis with eosinophilia, hepatosplenomegaly and secondary myelofibrosis. To clarify the clinical characteristics and prognosis of patients with Ph+ MPAL, we conducted a systemic literature research up to Apr. 2025 on PubMed and Embase databases with keywords and MeSH terms for mixed-phenotype acute leukemia, MPAL, Philadelphia chromosome, and Ph. As summarized in **Table 2** including 59 cases (20–36), patients with Ph+ MPAL are considered to have high WBC counts and primarily presented with B/myeloid phenotype (91.30%) (8, 15). There was no significant difference in sex, while a larger proportion of male patients were observed (54.24%) (7, 8, 20, 21). A slight tendency toward the p190 transcript (51.92%) was shown in patients with Ph+ MPAL. Analysis of 21 adult patients revealed no significant difference in clinical characteristics between patients with the p190 transcript and those with the p210 transcript (21). Whether different transcripts are involved in the phenotypic transition of MPAL needs further investigation given their different modes of signaling activation (37). Splenomegaly, hepatomegaly and lymph node enlargement were present in approximately 55.26%, 23.68% and 31.58% of patients, respectively. Additional CAs were shown in nearly 38.78% of cases. Deletion of chromosome 7 (-7), reported in 3/16 cases, appears to be a common CA and is considered an inferior prognostic biomarker, which is mostly a single anomaly in adults but is often accompanied with complex karyotype in children (15). Compared with those with AML or ALL, patients with biphenotypic acute leukemia more commonly present with complex karyotype and extramedullary infiltration (38), while extramedullary infiltration is relatively rare in patients with Ph+ MPAL with only two cases (32, 34). Concomitant *IKZF1* frameshift mutations have been reported in patients with B/M Ph+ MPAL (8), which are more frequent in ALL patients with complex karyotype and associated with a poor prognosis (39).

**Table 2:** Literature review of patients with Ph+ MPAL.

Ref.	Cases	Subtype	BCR::ABL1 Transcript	Age (yrs)	Sex (M)	WBC (×10^9)	Hemoglobin (mg/dL)	Platelet (×10^9)	Blast% in BM	LDH (IU/L)	Splenomegaly	Hepatomegaly	Lymph Node Enlargement	Additional CA or Karyotype	Mutations	Treatment	Outcomes	Extramedullary Infiltration
(21)	19	B/M	p190 (n=13)	31 (15-42)	7 (53.8%)	41.8 (3.0-681.0)	9.6 (4.5-14.6)	61 (18-150)	NA	NA	8/13	3/13	NA	4/13	NA	1. VDCP ± L-Asp or VDCAP 2. IM (61.9%) 3. allo-HSCT (4/16)	CR: 11/13 2-yrs OS: 23.0% 2-yrs RFS (%): 15.0%	NA
		B/M	p210 (n=6)	30 (18-50)	5 (83.3%)	151.1 (2.6-199.0)	8.8 (6.9-11.7)	69 (12-267)	NA	NA	5/6	1/6	NA	0/6	NA		CR: 4/6 2-yrs OS: 33.0% 2-yr RFS (%) 30.0%	NA
(35)	13	NA	p190 (n=4) p210 (n=9)	52 (16-80)	5 (38.46%)	24.7 (3.3-244.8)	NA	NA	NA	865 (195- 1947)	NA	NA	NA	6/13	NA	1. ALL-type (7/13) 2. AML-type (6/13) 3. allo-HSCT (8/13)	CR rates: 100% 5-yr OS: 55% 5-yr DFS: 46%	no
(20)	5	B/M (3/5) T/M (1/5) M/T/B (1/5)	NA	52 (38-61)	4 (80%)	58.6 (18–109)	9.02 (7.2-10.5)	86.2 (60-110)	NA	NA	NA	NA	NA	NA	NA	“7 + 3” regimen (Ara-C+DA)	CR: 2/5 OS: 4 (1-7) months	NA
(33)	4	B/M	p210	3.5	M	19.2	NA	45	NA	NA	mild	mild	yes	NA	NA		CR	NA
		B/M	p190	15	M	72	NA	11	NA	NA	no	no	yes	NA	NA	AML/ALL standard induction protocol+IM	not remission	NA
		B/M	p210	28	M	5.1	NA	381	NA	NA	no	no	no	NA	NA		not follow-up	NA
		B/M	p210	0.58	M	97.4	NA	118	NA	NA	no	no	no	NA	NA		CR	NA
(22)	4	B/M	p210	60	M	41.9	11.2	74.0	68.0	NA	yes	no	no	Complex karyotype without -7	NA	ALL-type+DA	MMR	NA
		B/M	p210	8	F	513.0	5.3	48.0	68.0	NA	yes	yes	yes	46,XX,t (9;22) (q34;q11.2)	NA	NA	NA	NA
		B/M	p210	10	F	376.0	6.7	33.0	48.0	NA	yes	yes	no	46,XX,t (9;22) (q34;q11.2)	NA	AML-type+IM	NA	NA
		B/M	p210	50	F	366.0	8.0	208.0	77.0	NA	yes	no	no	46,XX,t (9;22) (q34;q11.2)	NA	ALL-type+IM	NR	NA
(23)	2	B/M	p210	69	F	33.0	5.9	9.0	88.0	213	no	no	no	46,XX,t (9;22) (q34;q11.2)	NA	Pred+DA	MMR	NA
		B/M (therapy- related)	p190	69	F	160.0	11.3	259.0	99.0	503	no	no	no	46, XX, t (9;22) (q34;q11.2) [14] /46,idem,- 17,+mal[3]/46, XX[3].	NA	Pred+DA	MMR	NA
(24)	1	B/M	p190	85	M	12.9	11.2	70.0	52.0	NA	no	no	no	46, XX, t (9;22) (q34;q11.2)[14]/46,idem,-17,+mal	*TET2*	Decitabine+rituximab-mini-Hyper-CVAD+DA (also as consolidation chemotherapy)	MMR	NA
(25)	1	T/M (therapy- related)	p190	57	F	129	11.6	129.0	40.7	NA	NA	NA	NA	NA	NA	Refused chemotherapy	Death (3 months)	NA
(26)	1	B/M	NA	61	M	159.5	10.6	35.0	90.0	1607	no	no	no	45,XY,-7, t (9; 22) (q34;q11.2)	*c-MYC*	IM included therapy	Death (1.5 months)	NA
(27)	1	B/M	p190	54	M	49.7	11.8	303.0	70.0	NA	NA	NA	NA	46,XY, t (9; 22) (q34;q11.2)	NA	1st: IDA+CVP+IM 2nd: NI+lenalidomide	CR, Relapse CR	NA
(28)	1	B/M	p190	16	M	12.87	12.0	31.0	89.0	NA	yes	yes	yes	45,XY, dic (7; 9) (p11-13; p13), t (9; 22) (q34;q11) /46,XY	*PAX5::UBE2D4*	DVP+TKI	Relapse, Death (5 months)	NA
(29)	1	B/M	p190	71	F	26.5	10.9	13.3	71.0	1780	no	no	no	45,XX, -7, t (9;22) (q34;q11.2)	NA	IDA+IM/DA/NI (VP as maintenance therapy)	CR, Relapse	NA
(30)	1	B/M	NA	39	F	139.2	10.9	154.0	53.0	NA	no	no	no	46,XX, t (9; 22) (q34;q11.2)	NA	HyperCVAD/MTX- AraC+IM	NA	NA
(31)	1	B/M	p210	43	F	62.8	6.9	83.0	63.0	NA	no	no	no	46,XX,inv (9) (p12; q13), t (9; 22) (q34;q11.2)	NA	IDA+IM allo-HSCT after CR	Death (10 months)	NA
(32)	1	B/M (therapy- related)	p190	49	F	98.4	10.4	132.0	92.0	NA	NA	NA	NA	Complex karyotype without -7	NA	IDA+IM	MMR	yes
(34)	1	B/M	p190	61	M	150.1	6.7	37.0	diffuse infiltration	785	yes	no	yes	45,XY,-7, t (9;22) (q34;q11.2 )[19]/46,XY[1]	NA	Hyper-CVAD+DA allo-HSCT	CR	yes
(36)	1	B/M	p190	87	M	9.9	12.5	20.9	41.0	556	no	no	no	46,XY, t (9; 22) (q34;q11.2)	NA	Pred+DA/low dose-IM	HCR	NA
Presen t Article	1	T/M	p210	18	F	127.68	69	223	25.0	888	yes	yes	yes	46,XX,t (9;22) (q34;q11.2) [20]	*STAG2*	DVAP+IM/DA allo-HSCT	CR	no

M, male; F, female; VDC (A)P, vincristine+daunorubicin+cyclophosphamide+ (cytarabine)+prednisone; IDA, daunorubicin+cytarabine; Pred, prednisone; AraC, cytarabine; MTX, methotrexate; IM, imatinib; DA, dasatinib; NI, nilotinib; allo-HSCT, allogeneic hematopoietic stem cell transplantation; CR, complete remission; NR, not remission; OS, overall survival; RFS, recurrence free survival; DFS, disease free survival; MMR, major molecular response; HCR, hematologic complete remission; NA, not available.

In general, patients with MPAL are considered to have an inferior prognosis, especially in the older cohort (7, 40). A meta-analysis revealed that ALL-type therapy was statistically associated with a better CR rate and overall survival (OS) than AML-type regimen in the MPAL cohort (41). A multicenter retrospective study indicated that hyper-CVAD therapy (hyper-fractionated cyclophosphamide, vincristine, doxorubicin, dexamethasone) showed a significant effectivity and tolerance in patients with ALAL (42). Meanwhile, a study classified MPAL into AML-type and AML-type based on genome-wide methylation signatures, and claimed a better response when giving the lineage-matched therapy (43). B/T-MPAL has been found to share similar genetic characteristics with T-ALL rather than B-ALL, and was defined as a high-risk subgroup of ALL which could also benefit from ALL-based therapy (14). In contrast to subsequent consolidation chemotherapy, early allo-HSCT after initial CR may lead to a better prognosis (44). Prior to the TKI era, patients with Ph+ MPAL were considered to present dismal outcomes without allo-HSCT, especially female patients and those with WBC counts above 100×10^9^/L (7, 21). Nevertheless, TKI-combined therapy substantially improved the prognosis of patients with Ph+ MPAL, which is comparable to those with Ph+ ALL (21, 35, 45). In a small Japanese Ph+ acute leukemia cohort, there were no significant difference in the CR rate, 5-year OS or disease-free survival (DFS) rates between patients with Ph+ MPAL and those with Ph+ B-cell precursor (BCP)-ALL, when matched for influencing factors including age, sex, WBC counts, LDH levels, and the prevalence of additional CAs (35). A cancer registry analysis of 241 patients with MPAL revealed that the Ph+ cohort had a better prognosis than all other subtypes, whereas cases with *MLL* rearrangement were closely associated with poor OS (45). Among the specific cases listed in **Table 2**, six patients showed no response to TKIs or relapsed after remission. Primary resistance to imatinib was also observed in our present case. It currently unclear whether this is related to myelofibrosis, and further research is needed to explore the superiority of dasatinib over imatinib in such population. In elderly patients with Ph+ MPAL, low-dose TKIs in combination with prednisolone has been identified as a safe and effective therapeutic strategy (23, 36). Moreover, combination therapy with the BCL-2 inhibitor venetoclax is worth trying, as its great advantages in the treatment of elderly patients with AML (46). Given Ph as an adverse prognostic marker in AML, allo-HSCT at an early stage is recommended for eligible patients with myeloid-involved mixed-phenotype leukemia (18, 34). For patients who are not suitable for allo-HSCT, chimeric antigen receptor-T (CAR-T)-cell therapy may be considered as an alternative option in future research (47).

## Conclusion

In this study, we report a patient with Ph+ MPAL who received DVAP regimen combined with TKIs, followed by allo-HSCT, ultimately achieving hematologic and molecular CR. Through a literature review, similar to the overall MPAL population, the Ph+ subtype is more commonly observed in adult, male patients, and primarily presents as B/myeloid subtype. As an inferior prognostic marker, high WBC count has also been identified as one of the main characteristics of patients with Ph+ MPAL, while extramedullary infiltration is relatively rare. Chromosome -7 abnormalities have been occasionally reported in patients with Ph+ MPAL and are associated with a poor prognosis. ALL-type therapy is considered to more effective for patients with MPAL. In the TKI era, Ph+ MPAL has gradually become a subtype with a better prognosis. Meanwhile, novel therapeutic strategies are eagerly expected with the emergence of targeted therapeutic agents and immunotherapy.

## References

[1] Matutes E Morilla R Farahat N Carbonell F Swansbury J Dyer M. Definition of acute biphenotypic leukemia. Haematologica. (1997) 82:64–6. 9107085

[2] Rasekh EO Osman R Ibraheem D Madney Y Radwan E Gameel A. Acute lymphoblastic leukemia-like treatment regimen provides better response in mixed phenotype acute leukemia: a comparative study between adults and pediatric MPAL patients. Ann Hematol. (2021) 100:699–707. doi: 10.1007/s00277-020-04354-2, PMID: 33230570

[3] Weinberg OK Arber DA. How I diagnose acute leukemia of ambiguous lineage. Am J Clin Pathol. (2022) 158:27–34. doi: 10.1093/ajcp/aqac070, PMID: 35775438

[4] Vardiman JW Thiele J Arber DA Brunning RD Borowitz MJ Porwit A. The 2008 revision of the World Health Organization (WHO) classification of myeloid neoplasms and acute leukemia: rationale and important changes. Blood. (2009) 114:937–51. doi: 10.1182/blood-2009-03-209262, PMID: 19357394

[5] Bene MC Castoldi G Knapp W Ludwig WD Matutes E Orfao A. Proposals for the immunological classification of acute leukemias. European Group for the Immunological Characterization of Leukemias (EGIL). Leukemia. (1995) 9:1783–6., PMID: 7564526

[6] Khoury JD Solary E Abla O Akkari Y Alaggio R Apperley JF. The 5th edition of the world health organization classification of haematolymphoid tumours: myeloid and histiocytic/dendritic neoplasms. Leukemia. (2022) 36:1703–19. doi: 10.1038/s41375-022-01613-1, PMID: 35732831 PMC9252913

[7] Matutes E Pickl WF Van't Veer M Morilla R Swansbury J Strobl H. Mixed-phenotype acute leukemia: clinical and laboratory features and outcome in 100 patients defined according to the WHO 2008 classification. Blood. (2011) 117:3163–71. doi: 10.1182/blood-2010-10-314682, PMID: 21228332

[8] Yan L Ping N Zhu M Sun A Xue Y Ruan C. Clinical, immunophenotypic, cytogenetic, and molecular genetic features in 117 adult patients with mixed-phenotype acute leukemia defined by WHO-2008 classification. Haematologica. (2012) 97:1708–12. doi: 10.3324/haematol.2012.064485, PMID: 22581002 PMC3487445

[9] Khan M Siddiqi R Naqvi K. An update on classification, genetics, and clinical approach to mixed phenotype acute leukemia (MPAL). Ann Hematol. (2018) 97:945–53. doi: 10.1007/s00277-018-3297-6, PMID: 29546454

[10] Huang J Zhou J Xiao M Mao X Zhu L Liu S. The association of complex genetic background with the prognosis of acute leukemia with ambiguous lineage. Sci Rep. (2021) 11:24290. doi: 10.1038/s41598-021-03709-7, PMID: 34934076 PMC8692450

[11] Andrews C Tierens A Minden M. The genomic and biological complexity of mixed phenotype acute leukemia. Crit Rev Clin Lab Sci. (2021) 58:153–66. doi: 10.1080/10408363.2020.1829537, PMID: 33161794

[12] Hennawi M Pakasticali N Tashkandi H Hussaini M. Genomic landscape of mixed-phenotype acute leukemia. Int J Mol Sci. (2022) 23(19):11259. doi: 10.3390/ijms231911259, PMID: 36232559 PMC9569865

[13] Alexander TB Gu Z Iacobucci I Dickerson K Choi JK Xu B. The genetic basis and cell of origin of mixed phenotype acute leukaemia. Nature. (2018) 562:373–9. doi: 10.1038/s41586-018-0436-0, PMID: 30209392 PMC6195459

[14] Mi X Griffin G Lee W Patel S Ohgami R Ok CY. Genomic and clinical characterization of B/T mixed phenotype acute leukemia reveals recurrent features and T-ALL like mutations. Am J Hematol. (2018) 93:1358–67. doi: 10.1002/ajh.v93.11, PMID: 30117174 PMC8193761

[15] Manola KN. Cytogenetic abnormalities in acute leukaemia of ambiguous lineage: an overview. Br J Haematol. (2013) 163:24–39. doi: 10.1111/bjh.2013.163.issue-1, PMID: 23888868

[16] Rubnitz JE Onciu M Pounds S Shurtleff S Cao X Raimondi SC. Acute mixed lineage leukemia in children: the experience of St Jude Children's Research Hospital. Blood. (2009) 113:5083–9. doi: 10.1182/blood-2008-10-187351, PMID: 19131545 PMC2686179

[17] Al-Seraihy AS Owaidah TM Ayas M El-Solh H Al-Mahr M Al-Ahmari A. Clinical characteristics and outcome of children with biphenotypic acute leukemia. Haematologica. (2009) 94:1682–90. doi: 10.3324/haematol.2009.009282, PMID: 19713227 PMC2791935

[18] Dohner H Wei AH Appelbaum FR Craddock C DiNardo CD Dombret H. Diagnosis and management of AML in adults: 2022 recommendations from an international expert panel on behalf of the ELN. Blood. (2022) 140:1345–77. doi: 10.1182/blood.2022016867, PMID: 35797463

[19] Shah NP Bhatia R Altman JK Amaya M Begna KH Berman E. Chronic Myeloid Leukemia, Version 2.2024, NCCN clinical practice guidelines in oncology. J Natl Compr Canc Netw. (2024) 22:43–69. doi: 10.6004/jnccn.2024.0007, PMID: 38394770

[20] Atfy M Al Azizi NM Elnaggar AM. Incidence of Philadelphia-chromosome in acute myelogenous leukemia and biphenotypic acute leukemia patients: And its role in their outcome. Leuk Res. (2011) 35:1339–44. doi: 10.1016/j.leukres.2011.04.011, PMID: 21612824

[21] Wang Y Gu M Mi Y Qiu L Bian S Wang J. Clinical characteristics and outcomes of mixed phenotype acute leukemia with Philadelphia chromosome positive and/or bcr-abl positive in adult. Int J Hematol. (2011) 94:552–5. doi: 10.1007/s12185-011-0953-1, PMID: 22015493

[22] Choi W Kim M Lim J Han K Lee S Lee JW. Four cases of chronic myelogenous leukemia in mixed phenotype blast phase at initial presentation mimicking mixed phenotype acute leukemia with t(9;22). Ann Lab Med. (2014) 34:60–3. doi: 10.3343/alm.2014.34.1.60, PMID: 24422198 PMC3885775

[23] Takata H Ikebe T Sasaki H Miyazaki Y Ohtsuka E Saburi Y. Two elderly patients with philadelphia chromosome positive mixed phenotype acute leukemia who were successfully treated with dasatinib and prednisolone. Intern Med. (2016) 55:1177–81. doi: 10.2169/internalmedicine.55.5223, PMID: 27150875

[24] Jain P Tang G Huh YO Yin CC Zuo Z Pemmaraju N. Philadelphia-positive dimorphic blasts in mixed-phenotype acute leukemia with TET2 mutation. Am J Hematol. (2016) 91:647–8. doi: 10.1002/ajh.24295, PMID: 26799924 PMC4870100

[25] Yang D Cho SR Jung S Lee W Hwang HY Lee HS. A case of therapy-related acute leukemia with mixed phenotype with BCR-ABL1 after treatment of diffuse large B-cell lymphoma. Ann Lab Med. (2017) 37:166–8. doi: 10.3343/alm.2017.37.2.166, PMID: 28029006 PMC5203997

[26] Xu Z Padmore R Shier L Beaulieu Bergeron M. A rare case of acute leukemia of ambiguous lineage overexpressing C-MYC with monosomy 7 and Philadelphia chromosome. Ann Hematol. (2015) 94:1761–3. doi: 10.1007/s00277-015-2443-7, PMID: 26159563

[27] Lai B Mu Q Zhu H Wang Y Zhang Y Xu K. Durable remission in a patient of mixed phenotype acute leukemia with Philadelphia chromosome-positive treated with nilotinib and lenalidomide: A case report. Med (Baltimore). (2018) 97:e0294. doi: 10.1097/MD.0000000000010294, PMID: 29620650 PMC5902271

[28] Yu Y Zeng Z Xie J Lu Q Cai W Zhang R. Case report: the formation of a truncated PAX5 transcript in a case of ph-positive mixed phenotype acute leukemia with dic(7;9)(p11-p13;p13). Front Oncol. (2021) 11:703612. doi: 10.3389/fonc.2021.703612, PMID: 34513684 PMC8427297

[29] Kawajiri C Tanaka H Hashimoto S Takeda Y Sakai S Takagi T. Successful treatment of Philadelphia chromosome-positive mixed phenotype acute leukemia by appropriate alternation of second-generation tyrosine kinase inhibitors according to BCR-ABL1 mutation status. Int J Hematol. (2014) 99:513–8. doi: 10.1007/s12185-014-1531-0, PMID: 24532437

[30] Yong WL Yusof N Ithnin A Shuib S Tumian R Yousuf R. Mixed phenotype acute leukaemia with t(9,22), BCR-ABL1: A case report. Malays J Pathol. (2020) 42:469–76., PMID: 33361731

[31] Kim HN Hur M Kim H Ji M Moon HW Yun YM. First case of biphenotypic/bilineal (B/myeloid, B/monocytic) mixed phenotype acute leukemia with t(9;22)(q34;q11.2);BCR-ABL1. Ann Clin Lab Sci. (2016) 46:435–8., PMID: 27466307

[32] Cho JH Hur M Moon HW Yun YM Ko YS Kim WS. Therapy-related acute leukemia with mixed phenotype and t(9;22)(q32;q11.2): a case report and review of the literature. Hum Pathol. (2012) 43:605–9. doi: 10.1016/j.humpath.2011.07.010, PMID: 22036054

[33] Bhatia P Binota J Varma N Bansal D Trehan A Marwaha RK. A study on the expression of BCR-ABL transcript in mixed phenotype acute leukemia (MPAL) cases using the reverse transcriptase polymerase reaction assay (RT-PCR) and its correlation with hematological remission status post initial induction therapy. Mediterr J Hematol Infect Dis. (2012) 4:e2012024. doi: 10.4084/mjhid.2012.024, PMID: 22708039 PMC3375663

[34] Chan O Jamil AR Millius R Kaur R Anwer F. Mixed phenotype acute leukemia with t(9;22): success with nonacute myeloid leukemia-type intensive induction therapy and stem cell transplantation. Clin Case Rep. (2017) 5:435–9. doi: 10.1002/ccr3.2017.5.issue-4, PMID: 28396764 PMC5378833

[35] Shimizu H Yokohama A Hatsumi N Takada S Handa H Sakura T. Philadelphia chromosome-positive mixed phenotype acute leukemia in the imatinib era. Eur J Haematol. (2014) 93:297–301. doi: 10.1111/ejh.2014.93.issue-4, PMID: 24750307

[36] Okayama Y Takakuwa T Otomaru I Horiuchi M Miura A Araki T. Efficacy and safety of low-dose imatinib in an elderly patient with mixed phenotype acute leukemia with t(9;22)(q34;q11.2);BCR-ABL1. Clin Case Rep. (2021) 9:e04126. doi: 10.1002/ccr3.4126, PMID: 34026165 PMC8123726

[37] Cutler JA Tahir R Sreenivasamurthy SK Mitchell C Renuse S Nirujogi RS. Differential signaling through p190 and p210 BCR-ABL fusion proteins revealed by interactome and phosphoproteome analysis. Leukemia. (2017) 31:1513–24. doi: 10.1038/leu.2017.61, PMID: 28210003

[38] Xu XQ Wang JM Lu SQ Chen L Yang JM Zhang WP. Clinical and biological characteristics of adult biphenotypic acute leukemia in comparison with that of acute myeloid leukemia and acute lymphoblastic leukemia: a case series of a Chinese population. Haematologica. (2009) 94:919–27. doi: 10.3324/haematol.2008.003202, PMID: 19454497 PMC2704302

[39] Kirtek T Chen W Laczko D Bagg A Koduru P Foucar K. Acute leukemias with complex karyotype show a similarly poor outcome independent of mixed, myeloid or lymphoblastic immunophenotype: A study from the Bone Marrow Pathology Group. Leuk Res. (2023) 130:107309. doi: 10.1016/j.leukres.2023.107309, PMID: 37210875

[40] Shi R Munker R. Survival of patients with mixed phenotype acute leukemias: A large population-based study. Leuk Res. (2015) 39:606–16. doi: 10.1016/j.leukres.2015.03.012, PMID: 25858895

[41] Maruffi M Sposto R Oberley MJ Kysh L Orgel E. Therapy for children and adults with mixed phenotype acute leukemia: a systematic review and meta-analysis. Leukemia. (2018) 32:1515–28. doi: 10.1038/s41375-018-0058-4, PMID: 29550836 PMC7508489

[42] Duong VH Begna KH Kashanian S Sweet K Wang ES Caddell R. Favorable outcomes of acute leukemias of ambiguous lineage treated with hyperCVAD: a multi-center retrospective study. Ann Hematol. (2020) 99:2119–24. doi: 10.1007/s00277-020-04179-z, PMID: 32676733

[43] Takahashi K Wang F Morita K Yan Y Hu P Zhao P. Integrative genomic analysis of adult mixed phenotype acute leukemia delineates lineage associated molecular subtypes. Nat Commun. (2018) 9:2670. doi: 10.1038/s41467-018-04924-z, PMID: 29991687 PMC6039465

[44] Wolach O Stone RM. Mixed-phenotype acute leukemia: current challenges in diagnosis and therapy. Curr Opin Hematol. (2017) 24:139–45. doi: 10.1097/MOH.0000000000000322, PMID: 28099272

[45] Qasrawi A Ramlal R Munker R Hildebrandt GC. Prognostic impact of Philadelphia chromosome in mixed phenotype acute leukemia (MPAL): A cancer registry analysis on real-world outcome. Am J Hematol. (2020) 95:1015–21. doi: 10.1002/ajh.25873, PMID: 32419244

[46] Fowler-Shorten DJ Hellmich C Markham M Bowles KM Rushworth SA. BCL-2 inhibition in haematological Malignancies: Clinical application and complications. Blood Rev. (2024) 65:101195. doi: 10.1016/j.blre.2024.101195, PMID: 38523032

[47] Karsten H Matrisch L Cichutek S Fiedler W Alsdorf W Block A. Broadening the horizon: potential applications of CAR-T cells beyond current indications. Front Immunol. (2023) 14:1285406. doi: 10.3389/fimmu.2023.1285406, PMID: 38090582 PMC10711079

