# Supplementary material for: Philadelphia chromosome-positive mixed-phenotype acute leukemia: a case report and literature review
Source: Front Oncol. 2025 Jul 25;15:1623528. doi: 10.3389/fonc.2025.1623528 (PMC12331478; doi:10.3389/fonc.2025.1623528)
Supplement: Supplementary file 1 [file Table1.docx]

Table S1. Gene mutations for leukemia

| 17 Gene Mutations for AML | ASXL1, BCOR, CEBPA, DNMT3A, EZH2, FLT3, IDH1, IDH2, KIT, NPM1, RUNX1, SF3B1, SRSF2, STAG2, TP53, U2AF1, ZRSR2 |
| --- | --- |
| 7 Gene Mutations for ALL | ABL1, FBXW7, IKZF1, JAK1, JAK3, NOTCH1, TP53 |

Table S2. Fusion genes for leukemia

| 58 Fusion Genes for Leukemia | KMT2A::AFF1, KMT2A::MLLT6, KMT2A::ELL, RUNX1::RUNX1T1, RUNX1::MDS1/EVI1, PML::RARA, PRKAR1A::RARA, FIP1L1::RARA, TCF3::PBX1, EML1::ABL1, FIP1L1::PDGFRA, BCR::FGFR1, NuP98::NSD1, NuP98::KDM5A, KAT6A::CREBBP, KMT2A::MLLT4, KMT2A::EPS15, KMT2A::MLLT1, RUNX1::MDS1, RPN1::EVI1, ZBTB16::RARA, BCOR::RARA, NMP1::ALK, TCF3::HLF, ETV6::RUNX1, ETV6::PDGFRB, NuP98::HOxA9, NuP98::HOxC11, NuP98::RARG, MNX1::ETV6, KMT2A::MLLT3, KMT2A::MLLT11, KMT2A::PTD, RUNX1::EAP, SET::NUP214, NPM1::RARA, NABP1::RARA, NMP1::MLF1, SIL-TAL1, ETV6::ABL1, FUS::ERG, NuP98::HOxA11, NuP98::HOxD13, RBM15::MKL1, KMT2A::MLLT10, KMT2A::FOXO4, PICALM::MLLT10, RUNX1::MTG16, DEK::NUP214, STAT5b::RARA, NuMA1::RARA, CBFB::MYH11, BCR::ABL1, ETV6::PDGFRA, ZNF198::FGFR1, NuP98::HOxA13, NuP98::PMX1, CBFA2T3::GLIS2 |
| --- | --- |
